# The complete mitochondrial genome of *Melanotus cribricollis* (Coleoptera: Elateridae) and phylogenetic analysis

**DOI:** 10.1080/23802359.2019.1669085

**Published:** 2019-09-24

**Authors:** Yang Wang, Yang Liu

**Affiliations:** a College of Biology Pharmacy and Food Engineering, Shangluo University, Shangluo, Shaanxi Province, China;; b Key Laboratory of Resource Biology and Biotechnology in Western China (Ministry of Education) and College of Life Science, Northwest University, Xi’an, Shaanxi Province, China

**Keywords:** Insecta, molecular phylogeny, mitochondrial DNA., genetic diversity

## Abstract

We determined the complete mitogenome sequence of *Melanotus cribricollis* (Faldermann) (GenBank accession no. MK792748). The mitogenome is 15,908 bp in length, consisting of 13 protein-coding genes, 22 transfer RNA genes, two ribosomal RNA genes, and one non-coding control region. The overall nucleotide composition was 40.8% A, 31.5% T, 17.4% C, and 10.3% G, with 72.3% of AT, respectively. Phylogenetic analysis based on the 13 coding protein genes nucleotide sequences revealed that *M. cribricollis* clustered with the same genus species *M. villosus*, and the three genus *Melanotus* Eschscholtz, *Agriotes* Eschscholtz and *Adrastus* Eschscholtz had a close relationship.

Elaterids are among the most abundant beetles in many terrestrial habitats and some in larval form are destructive agricultural pests. Elateridae commonly known as click beetles contains approximately 10,000 species (Johnson 2002). *Melanotus cribricollis*, black integrally, widely distributed in China and East Asia (Jiang and Wang 1999). The sampled specimen was collected from the city of Shangluo, China (the geospatial coordinates: 33°51′57″N, 109°57′05″E) in April 2018. The specimen was stored in the Entomological specimen room of Shangluo University (voucher no. CO-2018230). Genomic DNA was extracted from muscle tissues using DNeasy DNA Extraction kit (Qiagen, Hilden, Germany). The complete mitochondrial DNA sequence of *M. cribricollis* was determined by Illumina HiSeq 2500 Sequencing System. In total, 5.8 G raw reads were obtained, quality-trimmed, and assembled using MITObim v 1.7 (Hahn et al. 2013). The genome was annotated using software GENEIOUS R8 (Biomatters Ltd., Auckland, New Zealand).

The complete mitochondrial genome of *M. cribricollis* was 15,908 bp in total length and had been deposited in GenBank database with an accession number MK792748. The overall base composition was 40.8% A, 31.5% T, 17.4% C, and 10.3% G, with an A + T ratio of 72.3%. The gene arrangement of *M. cribricollis* is found to be similar to most insect mitochondrial genomes (Wolstenholme 1992). The full mitochondrial genome contains 13 protein-coding genes (PCGs), 22 transfer RNAs (tRNAs), 2 ribosomal RNAs (rRNAs), and a putative control region (CR). Most PCGs of *M. cribricollis* have the conventional start codon for invertebrate mitochondrial PCGs (ATN), with the exception of *nad4* (GTG), *nad1* (TTG) and *cox1* (AAT), as the asparagine (AAT or AAC) are proposed to be the start codon for cox1 in suborder Polyphaga (Sheffield et al. 2008). Most of the PCGs terminate with the stop codon TAA or TAG, whereas *nad5*, *cox2*, and *cox3* end with the incomplete codon T. The 22 tRNA genes range from 63 bp (*trnC*) to 70 bp (*trnK*, *trnW* and *trnV*). Two rRNA genes (*rrnL* and *rrnS*) locate at *trnL1/trnV* and *trnV*/control regions, respectively. The lengths of the two rRNA genes (*rrnL* and *rrnS*) in *M. cribricollis* are about 1280 and 739 bp, with the A + T contents of 78.5 and 75.8%, respectively. The length of control region is 1281 bp with the AT content of this region is up to 79.8%.

To validate the phylogenetic position of *M. cribricollis*, 13 mitochondrial protein-coding genes sequences were extracted from the complete mitochondrial DNA sequences of 15 closely related taxa of Elateridae. The phylogenetic tree was constructed using the maximum-likelihood method through raxmlGUI 1.5 (Silvestro and Michalak 2012). Results showed that the family Elateridae is monophyletic, which was consistent with the previous studies (Douglas 2011; Lin et al. 2018) (Figure 1). The new sequenced species *M. cribricollis* got together with the same genus species *Melanotus villosus*, and *Melanotus* was sister to *Agriotes* and *Adrastus*, indicating the close relationship of these three genus. As conclusion, we obtained and described the complete mitochondrial genome of *M. cribricollis*, and the phylogenetic tree provided a reference for understanding the taxonomic status.

**Figure 1.**
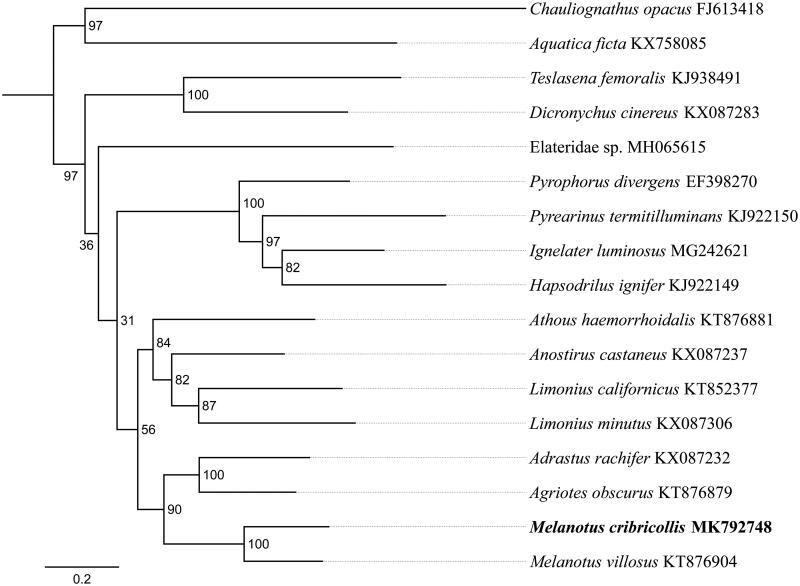
Phylogenetic relationships based on the 13 mitochondrial protein-coding genes sequences inferred from RaxML. Numbers on branches are Bootstrap values (BV).

## References

[CIT0001] Douglas H 2011 Phylogenetic relationships of Elateridae inferred from adult morphology, with special reference to the position of Cardiophorinae. Zootaxa. 2900:1–45.

[CIT0002] Hahn C, Bachmann L, Chevreux B 2013 Reconstructing mitochondrial genomes directly from genomic next-generation sequencing reads-a baiting and iterative mapping approach. Nucleic Acids Res. 41:e129. 23661685 10.1093/nar/gkt371 PMC3711436

[CIT0003] Jiang SH, Wang SY 1999 Economic click beetle faun-na of China (Coleoptera: Elateridae). Beijing: China Agriculture Press (Chinese).

[CIT0004] Johnson PJ 2002 Elateridae Vol. 2 In: Arnett RH, Thomas MC, Skelley PE, Frank JH, editors. American Beetles. Boca Raton, Florida, USA: CRC Press LLC; p. 160–173.

[CIT0005] Lin A, Zhao X, Song N, Zhao T 2018 Analysis of the complete mitochondrial genome of click beetle *Agriotes hirayamai* (Coleoptera: Elateridae). Mitochondr DNA B. 3:290–291. 10.1080/23802359.2018.1443041 PMC7800063 33474147

[CIT0007] Sheffield N, Song H, Cameron S, Whiting M 2008 A comparative analysis of mitochondrial genomes in Coleoptera (Arthropoda: Insecta) and genome descriptions of six new beetles. Mol Biol Evol. 25:2499–2509. 18779259 10.1093/molbev/msn198 PMC2568038

[CIT0008] Silvestro D, Michalak I 2012 RaxmlGUI: a graphical front-end for RAxML. Org Divers Evol. 12:335–337.

[CIT0009] Wolstenholme DR 1992 Animal mitochondrial DNA: structure and evolution. Int Rev Cytol. 141:173–216. 1452431 10.1016/s0074-7696(08)62066-5

